# Identification of stromal cell proportion-related genes in the breast cancer tumor microenvironment using CorDelSFS feature selection: implications for tumor progression and prognosis

**DOI:** 10.3389/fgene.2023.1165648

**Published:** 2023-07-27

**Authors:** Sicheng Guo, Yuting Ma, Xiaokang Li, Wei Li, Xiaogang He, Zheming Yuan, Yuan Hu

**Affiliations:** ^1^ Hunan Engineering & Technology Research Centre for Agricultural Big Data Analysis & Decision-Making, Hunan Agricultural University, Changsha, Hunan, China; ^2^ College of Life Sciences, University of Chinese Academy of Sciences, Beijing, China

**Keywords:** breast cancer, feature selection, cell proportion, dynamic trends, plasmablasts, prognostic marker

## Abstract

**Background:** The tumor microenvironment (TME) of breast cancer (BRCA) is a complex and dynamic micro-ecosystem that influences BRCA occurrence, progression, and prognosis through its cellular and molecular components. However, as the tumor progresses, the dynamic changes of stromal and immune cells in TME become unclear.

**Objective:** The aim of this study was to identify differentially co-expressed genes (DCGs) associated with the proportion of stromal cells in TME of BRCA, to explore the patterns of cell proportion changes, and ultimately, their impact on prognosis.

**Methods:** A new heuristic feature selection strategy (CorDelSFS) was combined with differential co-expression analysis to identify TME-key DCGs. The expression pattern and co-expression network of TME-key DCGs were analyzed across different TMEs. A prognostic model was constructed using six TME-key DCGs, and the correlation between the risk score and the proportion of stromal cells and immune cells in TME was evaluated.

**Results:** TME-key DCGs mimicked the dynamic trend of BRCA TME and formed cell type-specific subnetworks. The IG gene-related subnetwork, plasmablast-specific expression, played a vital role in the BRCA TME through its adaptive immune function and tumor progression inhibition. The prognostic model showed that the risk score was significantly correlated with the proportion of stromal cells and immune cells in TME, and low-risk patients had stronger adaptive immune function. IGKV1D-39 was identified as a novel BRCA prognostic marker specifically expressed in plasmablasts and involved in adaptive immune responses.

**Conclusions:** This study explores the role of proportionate-related genes in the tumor microenvironment using a machine learning approach and provides new insights for discovering the key biological processes in tumor progression and clinical prognosis.

## Graphical Abstract

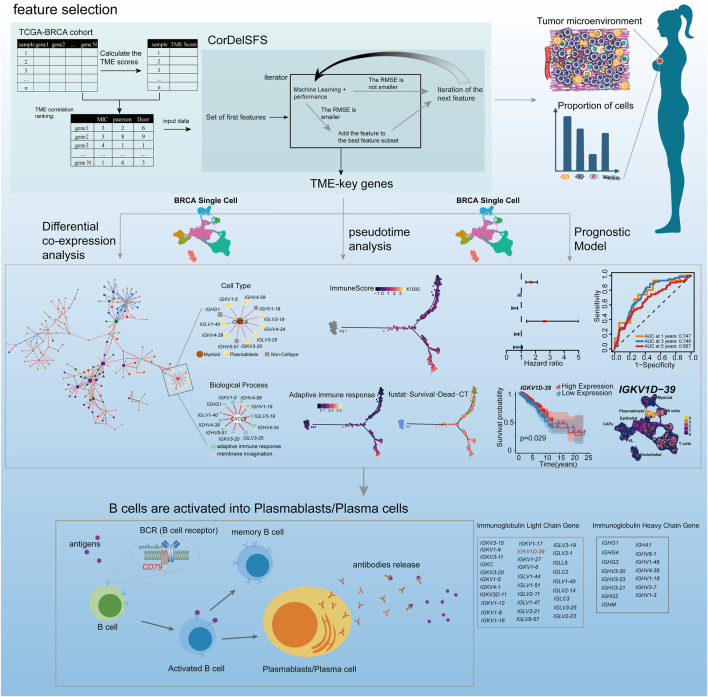

## 1 Introduction

Breast cancer (BRCA) is the most common cancer among women worldwide, accounting for 25.4% of all cancer cases in women and placing a heavy burden on both the health and finances of these patients (Ahmad, 2019). BRCA has a complex tumor microenvironment (TME), and the different cell types and altered gene expression patterns in the TME are all factors contributing to tumor heterogeneity that cannot be ignored. TME is a dynamic entity, characterized by changes in the types and quantities of various cell populations (Shalapour and Karin, 2015; Wang et al., 2020) that promote or inhibit tumor cell proliferation and metastasis throughout its evolution. Changes in stromal cells in the TME matter considerably in suppressing and promoting tumor metastasis during tumor evolution and metastasis initiation (Guo and Deng, 2018). For example, the co-evolution of malignant breast epithelial cells and their underlying mechanisms drive and support the occurrence of cancer-associated fibroblasts (CAFs) as a hallmark event in the development of most cancers (Roswall et al., 2018). The complement regulatory protein CD55 regulates the immune-promoting or immunosuppressive effects of tumor B cells by controlling the ICOSL + B cell production (Lu et al., 2020). However, the proportions of these important cell types within the TME were often overlooked. The composition of cell types within the TME varies between patients at different pathological stages (Yin et al., 2021), and the prognosis of the TME also differs in different states (Lohr et al., 2013; Germain et al., 2014; Goc et al., 2014; Giraldo et al., 2019). Additionally, the treatment measures for patients with different TME conditions are diverse (Ros and Vermeulen, 2018; Abou Khouzam et al., 2020; Shelton et al., 2021; Tiwari et al., 2022). Therefore, understanding the changes in cell types during breast cancer development can help us grasp the changing patterns of the TME in patients and thus provide targeted treatment for patients with different tumor microenvironments, improving their prognosis. Pseudo-temporal analysis can be used to simulate the development process of diseases and explore key molecular mechanisms (Gupta and Bar-Joseph, 2008; Tucker et al., 2015; Campbell and Yau, 2018). For example, PhenoPath has unsupervisedly simulated the disease trajectory of colorectal cancer and found that its trajectory fairly identified the immune contribution to the progression of colorectal cancer (Campbell and Yau, 2018). Therefore, pseudo-temporal analysis is helpful in analyzing the dynamic changes in the TME in breast cancer patients. In addition to changes in a cell type with tumor progression, at the transcriptomic level, some genes, such as the ferroptosis gene *ACSL4*/*GPX4* (Sha et al., 2021); the pyroptosis genes *GZMB*, *IL18*, *IRF1*, and *GZMA* (Wang et al., 2022); and the glycolysis-related genes *PGK1*, *SDHC*, *PFKL*, and *NUP43*, play a role in TME inhibition or promotion of tumor evolution and serve as prognostic markers (Zhang et al., 2021). However, these studies fail to assess the importance of a global perspective on tumor development and place no focus on the impact of dynamic changes in the cell type and proportion in the current TME on tumor development and prognosis. Furthermore, the regularity of dynamic changes in the proportion of different cell types during tumor development and the related genes is rarely reported.

Machine learning can efficiently identify potential target genes and can be used to identify genes related to changes in cell proportions in the BRCA TME. Many studies have developed feature selection algorithms for the removal of invalid and redundant features (Kong et al., 2009; Ekins et al., 2019; Mi et al., 2021), and machine learning models have been constructed for medical imaging diagnosis, cancer staging, and drug response prediction by selected genes or other biological variables (Curtis et al., 2012; Chiu et al., 2019; Liu et al., 2019). TME, as a dynamic network (Marx, 2013), features dynamically changing gene synergy, and individual genes cannot explain the biological processes of the TME in tumor progression (Tse and Kalluri, 2007; Im et al., 2021; Barkley et al., 2022). Therefore, we need to determine the connections between genes related to cell proportions. Yu et al. (2011) developed a differential co-expression analysis (DCEA) method to identify differentially co-expressed genes (DCGs) and differentially co-expressed gene pairs (DCLs) so as to precisely identify dynamic changes in the co-expression between gene pairs at different states. The bulk RNA cannot precisely determine whether the expression of key genes is driven by certain cell types (Li et al., 2022) or explain the altered gene co-expression relationships in relation to the proportion and function of cell types. However, single-cell transcriptomics (scRNA) can accurately localize the specific expression of genes in different cell types and the specific functions of each cell type (Grün and van Oudenaarden, 2015). The identification of genes affecting dynamic changes in the stromal cell proportion in the TME by machine learning and the construction of a cell type-specific co-expression network (CCEN) in the TME by DCEA combined with scRNA can explore changes in cell type-specific genes and co-expression patterns that drive changes in the cell proportion and function of different cell types during tumor progression, and thus facilitate the exploration of individual differences and prognosis.

Therefore, potential genes (TME-key genes) in the TCGA-BRCA cohort affecting changes in the stromal and immune cell proportion in BRCA TME were hereby identified based on an improved sequential forward selection (SFS) (Marcano-Cedeño et al., 2010) signature selection strategy. Furthermore, CCEN was constructed by DCEA and primary BRCA-scRNA to characterize the trajectory of stromal and immune cell proportions with tumor development, revealing the specific cell types in the TME and their underlying mechanisms. Finally, a TME-key-related prognostic model and new prognostic markers were constructed based on a series of prognostic analyses, including lasso regression, thereby providing new prognostic markers and new potential targets for immunotherapy and drug treatment.

## 2 Materials and methods

### 2.1 Data source

The data used for analysis included The Cancer Genome Atlas (TCGA)-gene expression matrix for breast cancer (TCGA-BRCA) (n = 1052), the Genotype-Tissue Expression (GTEx) database’s gene expression matrix for normal breast tissue (n = 179), and the single-cell data on primary BRCA (BRCA-scRNA) (Wu et al., 2021). A total of 130,246 single cells from BRCA-scRNA were downloaded from https://singlecell.broadinstitute.org/single_cell/study/SCP1039/. These cells underwent quality control and were annotated using the typical canonical lineage.

The validation dataset used in this study was obtained from multiple origins. First, additional nine normal breast transcriptome samples were included, consisting of four breast tissue samples from GSE31448 (Sabatier et al., 2011) and five breast tissue samples from Anton Buzdin et al.'s atlas of RNA sequencing profiles for normal human tissues (GSE120795) (Suntsova et al., 2019). These external datasets were used to validate the analysis results based on GTEx normal breast tissue and TCGA-BRCA data. Furthermore, the transcriptome data on 99 adjacent normal tissues from TCGA-BRCA were used to demonstrate the biological differences between adjacent normal and normal breast tissues. The samples of adjacent normal tissues, which lie between normal and tumor tissue, served as transitional data to validate the conclusions of this study. Finally, breast cancer samples from GSE31448 were employed to validate the prognostic model, and the Kaplan–Meier plotter (Lánczky and Győrffy, 2021) online website was used for the overall survival analysis (OS) of prognostic genes.

In addition, all transcriptome expression matrices were in the form of FPKM matrices. To remove batch effects and normalize the data, the “normalizeBetweenArrays” function from the R package “limma” was used.

### 2.2 The feasibility of jointly calculating differential genes from TCGA and GTEx

Due to the potential impact of tumor–stroma interactions on the transcriptional profiles of adjacent normal tissue in the tumor microenvironment, this study avoids the use of adjacent normal tissue from TCGA-BRCA samples as the control group for differential gene (DEG) analysis compared to TCGA-BRCA. Instead, large-scale transcriptome data from GTEx breast tissue are utilized to calculate DEGs alongside TCGA-BRCA.

The “normalizeBetweenArrays” function in R language is employed to correct batch effects between two datasets. Additionally, to demonstrate the differences in transcript levels between TCGA-BRCA’s cancer-adjacent tissue and normal breast tissue, we conducted sample clustering analysis based on principal component analysis (PCA) and Uniform Manifold Approximation and Projection (UMAP). This analysis was performed to assess the similarity between samples and ensure the authenticity and reliability of our research results.

A rank-sum test was used for DEG’s analysis of TCGA transcriptome matrix. Multiple testing corrections were carried out to control the overall error rate using the Benjamini–Hochberg false discovery rate (FDR), and an FDR < 0.05 and a |log2 fold change (FC)| > 2 were adopted as the cut-off criteria to identify the final DEGs.

### 2.3 Single-cell differential gene analysis

BRCA-scRNA was used to search for cell type-specific highly expressed genes and investigate the mechanism of action of related cell types on the TME. The R package “Seurat” was used for BRCA-scRNA analysis. The cellranger output file of BRCA-scRNA (Wu et al., 2021) was read into R and converted into Seurat objects, giving each cell of the Seurat object the corresponding cell type and information on the UMAP coordinate of the clusters. The “FeaturePlot” function determined the type of cells with high gene expression, the parameter order was set to TRUE, and the cells expressing the gene were placed at the top of the graph. The “FindAllMarkers” function calculated the DEGs of different cell types with default parameters, where logFC ≥ 0.25.

### 2.4 Forward non-kicking SFS signature selection for the identification of genes driving potential changes in cell proportions

A large amount of irrelevant information in features can lead to the degradation of model generalization performance in the case of too few samples and too many features in the dataset. An appropriate feature selection method can eliminate useless and redundant features, and capture the optimal subset of features beneficial for predicting the target information (predictor variables) so that the generalization performance of the model can be improved. Herein, the performance of feature selection was utilized to capture target information and genes that could be closely associated with changes in the stromal cell proportion. Specifically, a multi-step feature selection and model construction strategy (CorDelSFS) was proposed.

#### 2.4.1 Construction of the dataset

The DEGs were used as the original feature selection dataset, and to target the DEGs potentially associated with the TME, the R package “ESTIMATE” (Yoshihara et al., 2013) was used to calculate the TME scores for the entire TCGA tumor cohort. The immune cell relative proportion score (ImmuneScore) and stromal cell relative proportion score (StromalScore) were used as predictor variables for learning models in the feature selection strategy.

#### 2.4.2 Different correlation metrics for ranking the importance of genes to be selected

The maximum information coefficient (MIC) (Reshef et al., 2011), distance correlation coefficient (dcor) (Székely et al., 2007), and Pearson correlation coefficient were used to calculate the correlation between the expression of genes and the two TME scorings, with a higher correlation indicating a higher-level importance of the gene in the TME. Finally, the genes were ranked in accordance with their importance to determine the order of the gene input into the model.

#### 2.4.3 Improved SFS strategy

SFS (Marcano-Cedeño et al., 2010) is a classical wraparound feature selection method based on the principle of selecting one feature Xi at a time from the feature set X to join the feature subset S so that the loss function J (S + Xi, Y) can be maximized or minimized. In short, this method selects one feature at a time that makes J (S + Xi and Y) optimal. Furthermore, “forward” implies that the algorithm can only add features instead of removing them. Unimproved SFS algorithms may lead to redundancy due to their inefficiency in removing features. For example, the information space of feature A is a subset of features B and C. Suppose the SFS algorithm adds A, B, and C to the feature subset, the feature subset contains the redundant feature A, which will exert an impact on the prediction results of the model. Herein, an improved SFS algorithm was proposed. First, the features of TME importance ranking were input into the SFS model one by one to calculate the RMSE. Then, the algorithm used the RMSE as the judgment criterion to add useful features, following the principle of retaining useful features and rejecting useless features. Specifically, let the set of TME importance ranked features be X = [X1, X2, X3, … , Xi, … , Xn]; the number of features, n; the current set of introduced features, S; the number of introduced features, s; the number of unintroduced features, m; and the subset of unintroduced features, M (M = X-S); the introduction criterion and the loss function J (X and Y) is minimum. The introduction criterion for the s + 1 feature is

$$J\left({S+X}_{m+1},Y\right)<J\left({S+X}_{m},Y\right).$$ (1)

#### 2.4.4 Root-mean-square error (RMSE) as a loss function J

CorDelSFS predicts the dependent variable Y-pre using a linear regression model, with some error compared to the true Y. This error may be attributed to the performance of the learning machine or the noise of the trained dataset. To evaluate the merit of the training model and the feature genes, RMSE was thereby taken as the evaluation criterion for the model. The formula is as follows:

$$RMSE=\sqrt{\sum_{i=1}^{n}{\left(y_{test\_i}-{\hat{y}}_{test\_i}\right)}^{2}/n.}$$ (2)

#### 2.4.5 Comparison of other wraparound feature selection methods and machine learning models

To verify the superiority of CorDelSFS, other feature selection models were hereby used for comparison. From the feature selection strategy level, the compared feature selection methods included all without feature selection, the classical recursive feature elimination with cross-validation (RFEcv) and SFS without modification, the univariate filter with only relevance indicators, including MIC, Pearson, and dcor, and the method of inputting into SFS after sorting the correlation indicators (CorSFS). In terms of the level of embedded machine learning models, other machine learning methods embedded in the previously mentioned wrapped feature selection methods were used for the comparison with the hereby proposed feature selection models, including support vector machine regression (SVR), linear regression, random forest regression model (RF), decision tree (tree), and neural network (MLP).

### 2.5 Construction of the TME cell-specific differential co-expression network by integrating bulk RNA and single-cell RNA data

To investigate the unique co-expression patterns in the BRCA tumor microenvironment (TME), we performed differential co-expression analysis. However, differential co-expression networks based on bulk transcriptome can only measure the average level of gene expression changes in the tissue and cannot reveal the cell-type heterogeneity of gene expression. Single-cell transcriptomics, a technology that provides genome-scale molecular information at single-cell resolution, has been used to identify previously unknown cell types and associated markers (Treutlein et al., 2014; Zeisel et al., 2015; Shekhar et al., 2016). Therefore, we combined BRCA single-cell RNA sequencing data with differential co-expression analysis to assign cell-type labels to each gene node in the co-expression network and explore the TME cell-type heterogeneity in the network. The details are given in the following paragraphs.

#### 2.5.1 DCGL package to build differential co-expression networks

DCEA identifies DCGs by comparing altered gene expression patterns under different conditions. Herein, such clear differential co-expression relationships between genes were used for identifying key markers of disease (Chen et al., 2021) and key signaling pathway screening (Savino et al., 2020) among others.

The DCGL v2.0 (Liu et al., 2010) package in R was used to predict DCGs and differentially co-expressed linkages (DCLs), as well as to identify DCGs. The Pearson coefficient count (PCC) of any two genes, which reveals their co-expression relationship, was also calculated using DCGL v2.0 software. DCLs are hereby classified into three categories: a co-expression pattern present in normal samples but not in tumor samples, a co-expression linkage that is absent in normal samples while specifically present in BRCA samples, and a co-expression pattern present in normal samples but a complete reversal of this co-expression pattern in tumor samples.

BRCA-specific DCLs build co-expression networks. Herein, interaction information from DCGs and DCLs was input to Cytoscape software (Shannon et al., 2003) to establish the differentially co-expressed network. DCLs with absolute values of the correlation less than 0.3 (|cor|<0.3) were defined as irrelevant. DCLs with the correlation only in tumor patients (|cor_normal_|<0.3 and |cor_cancer_|≥0.3) were constituted as the BRCA TME-specific subnetwork. Finally, the DCLs of the subnetwork were filtered according to their correlation coefficient |cor_cancer_|≥0.5 and |cor_cancer_-cor_normal_|≥0.5 (|cor.diff|≥0.5), and displayed using Cytoscape.

#### 2.5.2 Markers of cell type-specific highly expressed genes in the network based on BRCA-scRNA

DEGs were calculated for each cell type of BRCA-scRNA. The “FindAllMarkers” function calculated the DEGs of different cell types with default parameters. DEGs in different cell types in BRCA-scRNA were filtered by logFC ≥ 1. If the gene node in the network is a DEG of certain cell types, then the gene node is labeled by this cell type, and the cell type with the highest logFC was taken as the cell type-specific marker for the gene node, in the case of the gene that is specifically highly expressed in different cell types. The cell-type specificity of gene nodes is marked with different colors in the network.

In addition, we also associated the TME-key enriched pathways with the cell types of gene nodes and mapped pathway activity in each cell in BRCA-scRNA to verify the relationship between enriched pathways and cell types at the single-cell level. Therefore, CCEN not only has differential co-expression information on genes but also mapping information on cell types and pathways. The impact of TME-key genes on the TME can be studied from multiple dimensions, including the gene level, cell level, and functional level. The Metascape website was used for pathway enrichment analysis.

Gene Ontology (GO) and KEGG pathway analyses were performed using the Metascape bioinformatics tool (http://metascape.org) (Zhou et al., 2019), and only terms with *p* values ≤ 0.05, minimum counts ≥ 3, and enrichment factors ≥ 1.5 were considered significant.

### 2.6 Area under curve (AUC) of ROC for gene set activity

The R package AUCell was used to calculate gene set enrichment scores, and the “area under the curve” (AUC) was adopted to calculate whether a subset of the input gene set was enriched in expressed genes in each sample. The distribution of AUC scores across all samples made it possible to explore the relative expression of features. Given that the scoring method was based on ranking, AUCell was independent of the gene expression units and normalization procedures.

### 2.7 Trajectory analysis

Pseudo-temporal analysis is a method of mapping high-dimensional molecular data to a series of one-dimensional quantities called pseudo-time. These pseudo-time measurements quantify the relative progression of each individual in the biological process of interest, such as disease progression or cell development, allowing us to understand the (pseudo) temporal behavior of measured features without explicit time-series data. All pseudo-temporal analyses include three important pieces of information: 1) the key genes, which are the result of feature selection, 2) the pseudo-time, which is a one-dimensional ordering space, and 3) the ordering, which represents the evolutionary trajectory of the study object. Therefore, the selection of key genes for pseudo-time analysis is crucial as it can directly affect the meaning of the ordering results.

We designed the CorDelSFS feature selection algorithm to identify genes related to changes in cell-type proportions in the breast cancer tumor microenvironment (TME) and further screened for breast cancer-specific co-expressed genes through differential co-expression analysis. These genes were used as input features for pseudo-temporal analysis to ensure that the final ordering results of the samples reflect the dynamic changes in the TME.

Specifically, based on the expression of 101 TME-key DCGs in the transcriptomic data from TCGA-BRCA patients and normal breast tissue of GTEx in a proposed time series, trajectory analysis was performed using the R package “Monocle2” (v2.18.0), which was run with GTEx as the reference starting point and the function “orderCells.” In addition, the “plot_genes_branched_heatmap” function was used to plot the heatmap of genes associated with changes in cell proportions along the differentiation trajectory.

In the end, we validated the developmental trajectory of the tumor microenvironment (TME) by utilizing external datasets from nine normal breast tissues and 99 TCGA-BRCA adjacent tissues. These samples were merged into a transcriptional matrix with GTEx normal breast tissues and TCGA-BRCA samples to reconstruct a pseudo-time trajectory, which served as the validation trajectory. We compared the relative positions of different sample sets, including normal breast tissue samples from various sources, TCGA adjacent tissue samples, and TCGA-BRCA samples, along the pseudo-time trajectory.

### 2.8 Determination of genes in the TME-key DCGs closely related to the prognosis in BRCA

The TME-key DCGs in BRCA were analyzed using Cox regression and the LASSO technique for their prognostic significance.

To select genes that contribute to the prognosis of BRCA, univariate Cox regression was first performed, with *p*-values less than 0.05 indicating statistical significance for genes. Genes having the biggest effects on the prognosis of BRCA were identified using the LASSO approach with an L1 penalty. By reducing the number of indicators with a final weight of non-zero and the regression coefficient, an L1 penalty was applied by LASSO to identify indicators contributing the most (Tibshirani, 1996). Furthermore, the glmnet package in R was hereby used to perform LASSO and thus reduce the number of genes using 1000 iterations and 10-fold cross-validations. The following related parameters were chosen: cv = 10 and maxiter = 1000. After 1,000 iterations of LASSO, the ability of the associated gene to predict the prognosis became stronger, and the non-zero coefficient was higher. Following the incorporation of the chosen genes into a multivariate Cox regression model, forward selection and backward removal were used to identify the gene set with the best prognostic value for BRCA.

### 2.9 Establishment and validation of a prognostic model

The gene set identified using the multivariate Cox regression was adopted to construct a prognostic model. The prognostic score formula was set up as follows: Risk Score = (a1 * TNFRSF14 expression level) + (a2 * SUSD3 expression level) + (a3 * COX7A1 expression level) + (a4 * ROBO3 expression level) + (a5 * FBLN5 expression level) + (a6 * IGKV1D-39 expression level). The median was used as a cutoff to distinguish between the high-risk and low-risk BRCA patients having survival data, while K–M curves and ROC curve analyses were used to assess the accuracy of the prognostic model in making predictions.

### 2.10 TME stromal cell scoring and analysis of the level of immune cell infiltration

The level of immune cell infiltration was calculated using the R package “MCPcounter,” which predicted the abundance of 10 cell populations from transcriptome profiles (CD3^+^ T cells, CD8^+^ T cells, CTLs (cytotoxic lymphocytes), NK (natural killer) cells, B lymphocytes, monocyte lineage cells, bone marrow dendritic cells, neutrophils, endothelial cells, and CAFs) (Becht et al., 2016) as continuous variables.

Then, risk score and prognostic marker expression were divided into the high and low groups according to the median values. The Wilcoxon rank-sum test was performed to compare the differences in cell infiltration levels, ImmuneScore, and StromalScore between the high and low groups.

Correlations between risk score, gene expression, infiltration levels of different cell types, immune inhibitor, and immune stimulator were calculated using the Pearson correlation coefficient (*p* < 0.05).

### 2.11 GSEA pathway enrichment

The samples were divided into two groups according to the expression of genes. All genes in the two groups were sorted by logFC, and the enrichment of the gene sets was calculated using GSEA.

GSEA pathway enrichment was performed using the function “GSEA” from the R package “clusterProfiler,” and the pathway database was downloaded from the GO database as “c5. go.v7.4. symbols”. Pathways of GOBP were selected, and the top five pathways with *p* < 0.05 and the highest NES values were selected. In addition, the high- and low-risk groups of the samples were taken by the GSEA of the prognostic model as the grouping in the calculation of the ranking.

## 3 Results

### 3.1 Machine learning identifies genes associated with stromal cell and immune cell proportions

**FIGURE 1:**
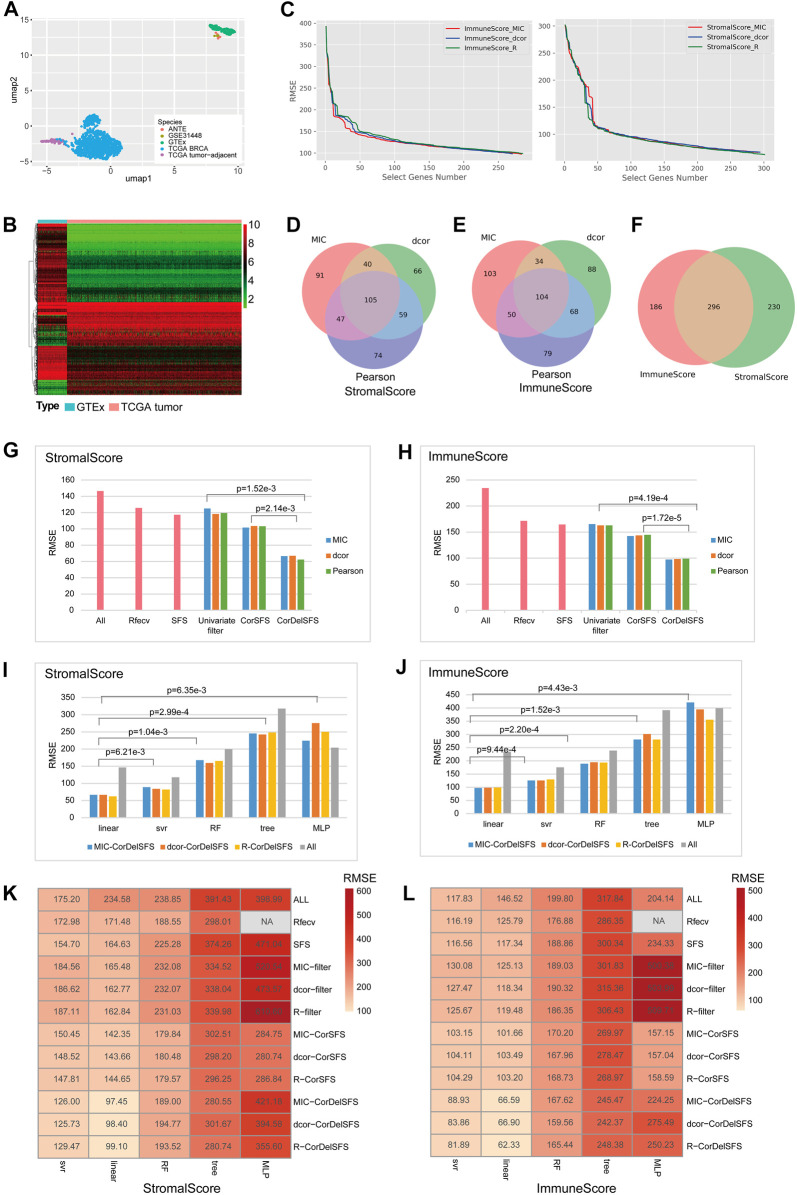
CorDelSFS filtering of the TME-key gene and model comparison: **(A)** UMAP plot for sample clustering. PCA and UMAP were utilized to demonstrate the similarity between samples. The samples were sourced from TCGA-BRCA, TCGA-BRCA adjacent normal tissue, GTEx normal breast tissue, ANTE database normal breast tissue, and GSE31448 normal breast tissue. **(B)** Heatmap showing the expression of DEGs in normal samples (GTEx) and BRCA samples (TCGA-BRCA). **(C)** The RMSE of the learning model during feature selection is reduced. MIC, dcor, and Pearson are three different indicators of gene importance ranking. ImmuneScore and StromalScore are the predictor variables. **(D,E)** The Venn diagram represents the overlap of the subset of genes screened by CorDelSFS for three correlation (MIC, dcor, and Pearson) rankings. **(D)** StromalScore as the predictor variable. **(E)** ImmuneScore as the predictor variable. **(F)** The Venn diagram represents the intersection of subsets of genes screened by CorDelSFS with StromalScore and ImmuneScore as predictor variables, with a total of 296 intersecting genes (TME-key genes). **(G,H)** Comparison of heuristic feature selection methods, including all DEGs (ALL), RFECV SFS, the univariate filter with only relevance indicators, including MIC, Pearson, and dcor, and the method of inputting into SFS after sorting the correlation indicators (CorSFS). All feature selection methods have embedded linear learning models. The RMSE of CorDelSFS is lower than other feature selection methods. **(G)** StromalScore as the predictor variable. **(H)** ImmuneScore as the predictor variable. **(I,J)** Comparison of learning model performance in feature selection methods, including linear, SVR, RF, tree, and MLP, with a lower RMSE in the linear method. **(I)** StromalScore as the predictor variable. **(J)** ImmuneScore as the predictor variable. **(K,L)** Heatmap showing model performance comparison of feature selection methods combined with machine learning. CorDelSFS (MIC-CorDelSFS, DCOR-CorDelSFS, and R-CorDelSFS) shows the best performance. Feature selection methods include all DEGs (ALL), RFECV, SFS, and the univariate filter with only relevance indicators, including MIC (MIC filter), Pearson (Pearson filter), and dcor (DCOR filter), and the method of inputting into SFS after sorting the correlation indicators (MIC-CorSFS, Pearson-CorSFS, and DCOR-CorSFS), and CorDelSFS. The shade of red indicates the RMSE value, and the lighter red indicates the lower RMSE and better model performance **(K)** with StromalScore as the predictor variable and **(L)** ImmuneScore as the predictor variable.

The graphical abstract presents an overview of the entire analytical process of the study (Graphical Abstract). First, based on the clustering analysis of tumor samples, adjacent normal samples, and normal breast samples, it was demonstrated that the GTEx normal samples formed a distinct cluster together with nine samples from two additional external normal datasets. They were completely separated from the TCGA-BRCA samples and adjacent data (Figure 1A). Additionally, the TCGA-BRCA adjacent tissue samples formed a separate cluster and were located closer to the TCGA-BRCA tumor samples, indicating the influence of tumor cells on the adjacent tissue. Therefore, GTEx normal breast tissue was utilized as the control group to calculate differentially expressed genes with BRCA, and two additional normal external datasets were used for subsequent result validation. A total of 930 DEGs (647 downregulated and 283 upregulated) were identified between TCGA-BRCA patients and GTEx normal breast tissue based on FDR<0.05 and logFC>2 thresholds (Figure 1B; Supplementary Table S1). Furthermore, we identified genes related to StromalScore and ImmuneScore among the 980 DEGs using CorDelSFS, a novel feature selection method integrated in this study for identifying genes associated with changes in cell proportions.

The process was followed by a decrease in RMSE as useful genes were retained (Figure 1C). Finally, the MIC-CorDelSFS, DCOR-CorDelSFS, and Pearson-CorDelSFS models based on StromalScore and ImmuneScore filtered 291, 294, and 301; and 283, 270, and 285 genes, respectively. The loss function RMSE of CorDelSFS was significantly lower than the full range of DEGs (Table 1). The correlation metrics presented their own characteristics (Rudra et al., 2017). In StromalScore-based CorDelSFS, MIC-CorDelSFS identified 91 unique genes, dcor-CorDelSFS had 66 genes, and Pearson-CorDelSFS contained 74 genes (Figure 1D). Meanwhile, in the ImmuneScore-based CorDelSFS, the MIC-CorDelSFS identified 103 unique genes, the dcor-CorDelSFS had 88 genes, and the Pearson-CorDelSFS contained 79 genes (Figure 1E). To this end, it could reasonably be claimed that different correlation algorithms could identify different correlations. Then, the gene sets of the correlation metrics were combined to reduce the loss of TME information. Finally, the intersection of the StromalScore and ImmuneScore gene subsets was taken to screen TME-key genes, and a total of 296 TME-key (TME-key) genes were successfully screened (Figure 1F; Supplementary Table S2).

**TABLE 1:** CorDelSFS screening signature genes and their error assessment.

		Number	RMSE
StromalScore	MIC	291	97.446
	dcor	294	98.404
	Pearson	301	99.098
	All	-	234.584
ImmuneScore	MIC	283	66.588
	dcor	270	66.897
	Pearson	285	62.325
	All	-	146.521

*Note:* “All” indicates that all genes were entered into the model for prediction.

To verify the efficacy of the selection strategy, CorDelSFS was compared with other strategies (Figures 1G, H). The selection strategies were divided into three categories, i.e., the unmodified classical RFECV and SFS, methods to filter genes using only correlation metrics, and SFS without correlation metric ranking. The RMSE of CorDelSFS is significantly lower than that of other methods, and the results have statistical significance as tested by the paired *t*-test. Therefore, CorDelSFS is considered to be significantly superior to other feature selection methods. In addition, the classical linear model with good robustness was hereby used as a training machine within CorDelSFS to evaluate the validity of each input gene. The linear regression model was compared with other learning machines, such as neural networks, support vector machines, and random forest regression models. The results still show that the linear model is slightly better than the support vector machine model and significantly better than the other models (Figures 1I, J). This result was also subjected to paired *t*-test analysis, demonstrating statistical significance.

Finally, all the previously mentioned training machines, feature selection strategies, and relevance metrics were combined, involving a total of 59 combinations, and the RMSE of all the combined models was calculated. The results show that among all the selection strategies, MIC-CorDelSFS, Dcor-CorDelSFS, and Pearson-CorDelSFS have the smallest test-set RMSE (Figures 1K, L).

### 3.2 Cell type-specific differential co-expression networks and TME dynamic changes

**FIGURE 2:**
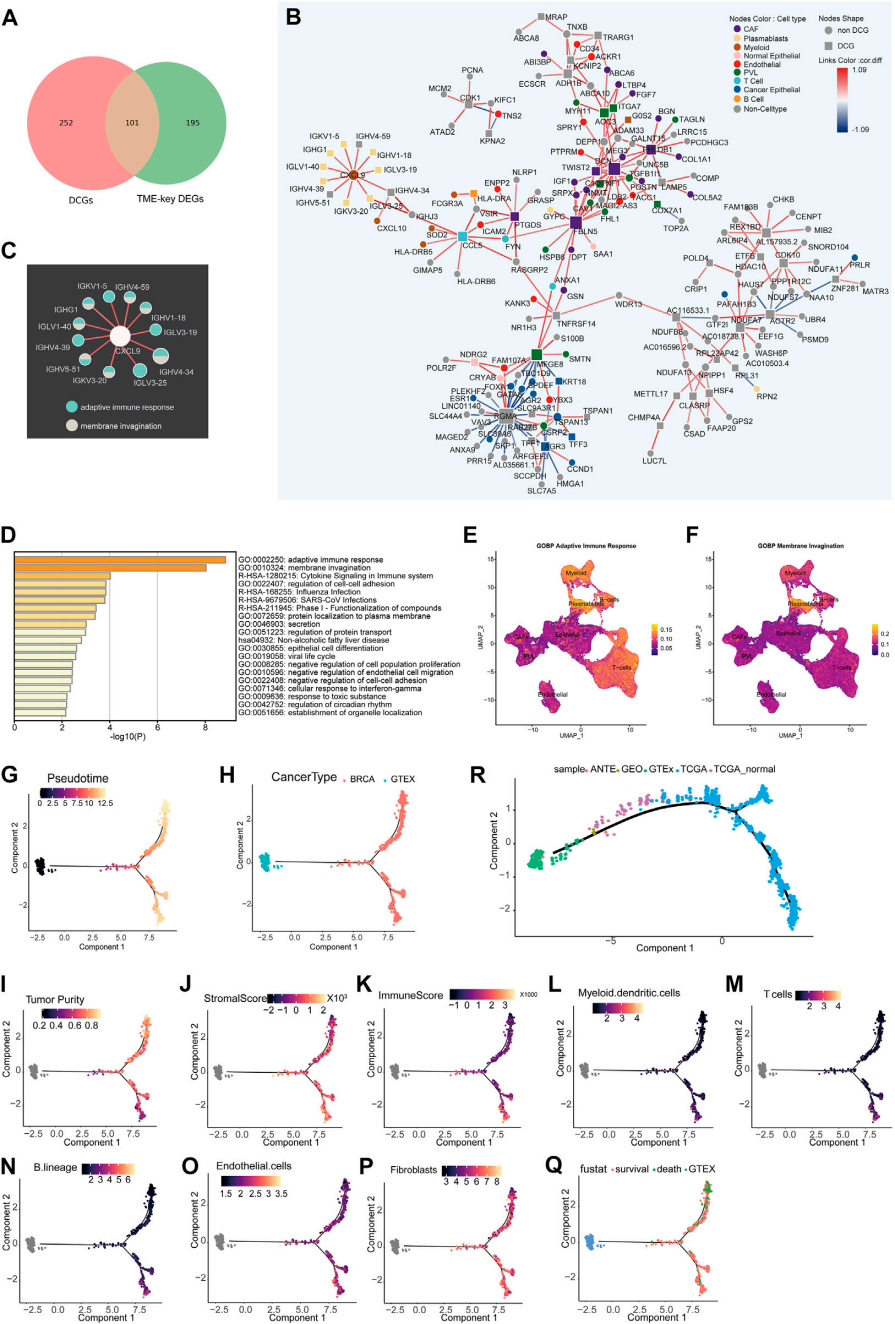
Differential co-expression network analysis of the TME and inference of state trajectories: **(A)** Venn diagram showing that TME-key genes contain 101 DCGs. **(B)** Specific occurrence of CCEN in BRCA TME (cor.diff≥0.5). The node shape indicates whether the gene is a DCG. The color of the node indicates the gene’s cell-type specificity. The color of the links indicates the difference in the correlation (cor.diff) compared to the normal control; red indicates the positive correlation, blue indicates the negative correlation, and the color shade indicates the size of the cor. diff value. **(C)** Pathway specificity of IG gene subnetworks. The color of the node represents pathways. **(D)** Metascape pathway enrichment analysis of 101 genes. **(E)** Scoring of the AUC activity of adaptive immune responses on BRCA-scRNA. **(F)** Scoring of the AUC activity of membrane invagination on BRCA-scRNA. **(G–Q)** The continuous change in the expression pattern of 101 TME-key DCGs simulates the continuous change in the TME state by the proposed time series analysis. The TME trajectory differentiates into two branches. The upper branch indicates the direction to tumor cells. The lower branch indicates the direction to stromal cells and immune cells. **(G)** Simulated time-series (Pseudotime) value of the differentiation trajectory. **(H)** Demonstration of the trajectory of TCGA-BRCA samples and GTE breast normal tissue. **(I–K)** Tumor purity **(I)**, stromal cell scoring **(J)**, and immune cell scoring **(K)** of TCGA-BRCA samples calculated using ESTIMATE. **(L–P)** Relative proportion of different types of infiltrating cells, including T cells **(L)**, B lineage **(M)**, myeloid dendritic cells **(N)**, fibroblasts **(O)**, and endothelial cells **(P)**, as calculated by MCPcounter. **(Q)** Clinical survival status of BRCA patients. **(R)** Trajectory of validation. Based on the pseudo-temporal trajectories of tumor tissue, adjacent tissue, and normal breast tissue, we validated the stability of constructing TME differentiation trajectories using 101 TME-key DCGs.

The tumor microenvironment is a dynamic network (Im et al., 2021). The co-expression patterns of genes and the proportion of each cell type in the TME are in the dynamic change as the tumor develops. Herein, a total of 101 DCGs (TME-key DCGs) and 100,258 associated DCLs (Supplementary Tables S3, S4) were identified from TME-key genes (Figure 2A). Pathway enrichment results show that 101 TME-key DCGs are mainly enriched to pathways such as adaptive immunity, membrane invagination, cell adhesion, cell junctions, and negative regulation of cell proliferation (Figure 2D).

The BRCA TME-specific gene co-expression pattern determines the biological mechanisms specific to BRCA TME, such as angiogenesis and stronger immune response. Thus, TME-specific CCEN was further constructed using co-expression patterns specific to the disease state, and the cell types of node genes in the network (Figure 2B) were mapped to analyze the dynamics of TME-key DCGs and the roles they played in the TME. There was a certain pattern in the distribution of genes marked by different cell types in the co-expression network in BRCA TME. In the network, genes were specifically expressed by immune class cells and non-immune class stromal cells form tight sub-networks, respectively. Genes specific to immune cells such as myeloid, T cells, B cells, and plasmablasts were co-expressed, while those specific to non-immune classes of stromal cells such as PVL, CAFs, and endothelial were more closely linked. The genes enriched in adaptive immune response and membrane invasion were mainly derived from the plasmablast-specific IG gene subnetwork (Figure 2C) encoding immunoglobulin components, and the AUC activities of the two pathways were also the highest in plasmablasts. Other immune cell types also had a higher activity of adaptive immune response pathways (Figures 2E, F).

To investigate the global changes in the stromal cell proportion, a trajectory analysis was performed by integrating GTEx and TCGA-BRCA samples and using the expression of 101 TME-key DCGs. The trajectory analysis mapped the expression of the 101 TME-key DCGs to a one-dimensional space to simulate the dynamic processes of stromal and immune cell proportions during tumor development (Figure 2G). The TME differentiation trajectory begins with normal breast tissue and differentiates into two major branches over time (Figure 2H). The upper branch shows an increase in BRCA tumor purity and a decrease in the TME stromal score with simulated time, indicating a direction favorable for the development of BRCA cancer cells (Figure 2I). The lower branch shows a higher TME stromal score and lower tumor purity, indicating a direction favorable for the survival of stromal cells (Figures 2J–P; Supplementary Figure S1). Furthermore, patients at the end of the lower branch had a lower mortality rate (Figure 2Q).

To validate the authenticity of the trajectory of changes in cell proportions, we included nine normal breast tissue samples and TCGA-BRCA adjacent tissue samples as an external validation dataset. We reconstructed a pseudo-temporal trajectory as the validation trajectory. The results showed that the shape of the validation trajectory closely resembled the original trajectory (Figure 2R). Normal tissues from different data sources were positioned closer to the starting point of the trajectory, while adjacent tissues occupied the “mid-transition zone” of the trajectory, and tumor tissues predominantly clustered along the trajectory branches. These results suggest that the cell proportion trajectory constructed based on the expression patterns of 101 TME-key DCGs is robust and not affected by data batches. It further suggests that tumor cells have a non-negligible impact on the surrounding tissue, and therefore, adjacent tissue cannot be considered normal tissue directly.

**FIGURE 3:**
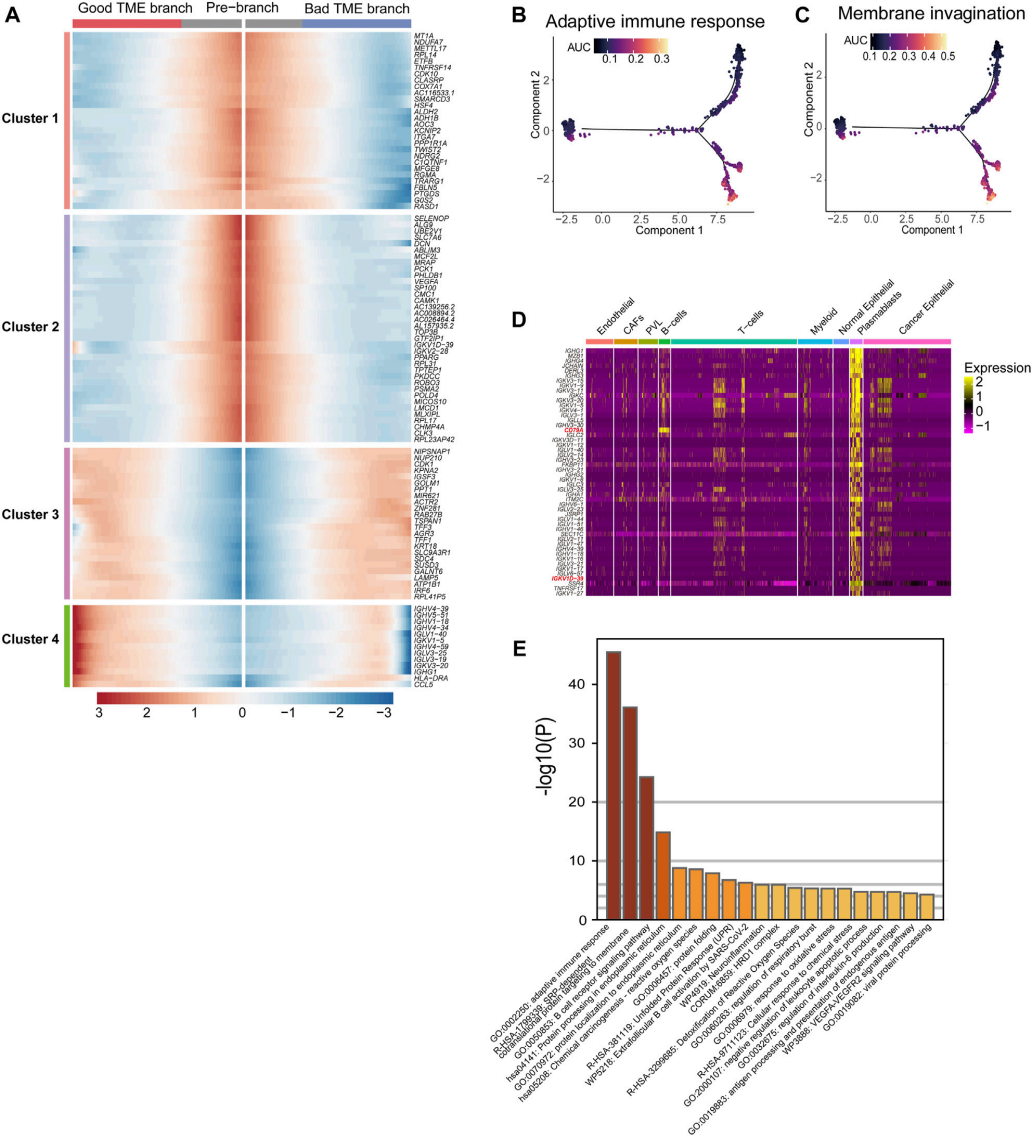
Differential genes in BRCA-scRNA in plasmablasts and pathway enrichment analysis: **(A)** Heatmap showing the expression of 101 TME-key DCGs differentiated from the origin (GTEx) along different branches. **(B,C)** Scoring of AUC activity in TCGA-BRCA and GTEx normal breast tissue for adaptive immunity **(B)** and the membrane invagination pathway. **(C)**. **(D)** Heatmap showing the expression of the top 50 DEGs upregulated in plasmablasts. **(E)** Pathway enrichment analysis of the top 200 DEGs upregulated in plasmablasts.

With the passage of pseudo-time, the expression of 101 TME key DCGs showed varying degrees of changes in two branches, among which the plasma cell-specific IG gene (cluster4) had opposite expression patterns in two different branches (Figure 3A). The IG gene was expressed higher in the lower branch, and the IG gene-related endocytosis and adaptive immune pathways had higher activity in the lower branch (Figures 3B, C). The plasmablast-specific IG gene co-expression network matters considerably in the environmental interactions and immune function of the BRCA immune microenvironment, influencing the trajectory of TME development and the survival of BRCA patients.

Furthermore, the top 50 differentially upregulated genes in plasmablasts in BRCA-scRNA contained many genes encoding antibody-like immunoglobulin light and heavy chains (IG genes) (Figure 3D). Pathway enrichment analysis of plasmablast DEGs shows that plasmablasts are mainly involved in adaptive immune response, SRP-dependent co-translational protein targeting to the membrane, B-cell receptor signaling pathway, etc. (Figure 3E), and that they play a role in the TME by synthesizing immunoglobulins to resist tumor cells and stop the progression and metastasis of BRCA.

### 3.3 Prognostic model construction and identification of prognostic markers in TME-key DEGs

**FIGURE 4:**
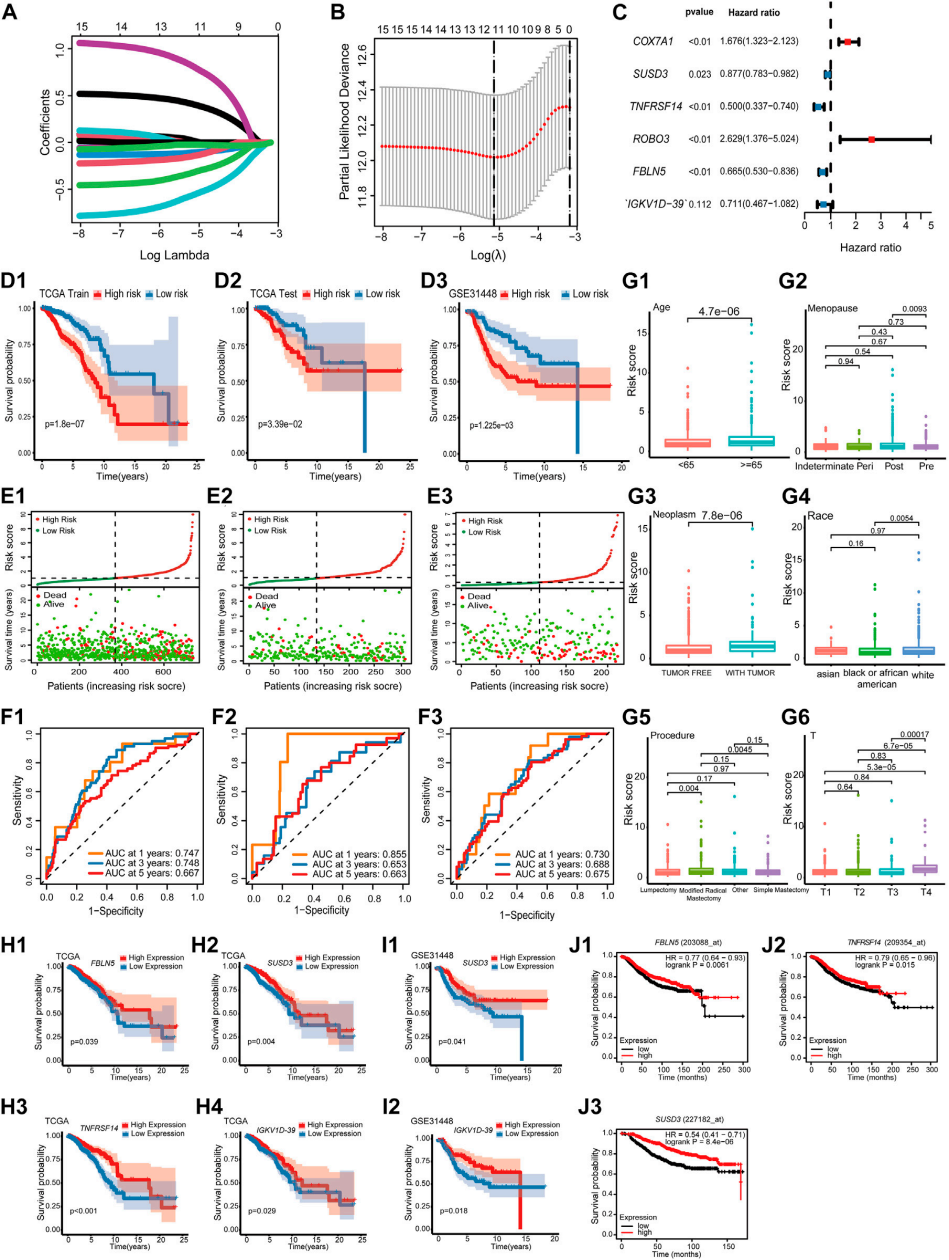
Prognostic model construction and identification of prognostic markers. **(A)** LASSO coefficient profiles. **(B)** Using 10-fold cross-validation based on the OS minimum criterion, the tuning parameters (lambda) in the LASSO model are chosen. **(C)** Forest map indicating independent predictors of prognosis in BRCA. **(D)** Analysis of OS survival in high- and low-risk patients. **(D1)** TCGA training cohort. **(D2)** TCGA test cohort. **(D3)** GSE31448 cohort. **(E)** Distribution of risk scores and OS of the TCGA training cohort (**E1**), TCGA test cohort **(E2)**, and GSE31448 cohort **(E3)**. **(F)**. Validation of the prognostic value of the prognostic index at 1, 3, and 5 years in TCGA training cohort **(F1)**, TCGA test cohort **(F2)**, and GSE31448 cohort **(F3)** using survival-dependent ROC curves. **(G)** The risk score was significantly correlated with age **(G1)**, menopause status **(G2)**, person neoplasm cancer status **(G3)**, race (**G4**), breast carcinoma surgical procedure name **(G5)**, and pathologic T-stage **(G6)**. **(H)** Validation of OS survival analysis of FBLN5 **(H1)**, SUSD3 **(H2)**, TNFRSF14 **(H3)**, and IGKV1D-39 **(H4)** in TCGA-BRCA. **(I)** Validation of OS survival analysis in the GSE31448 cohort of SUSD3 **(I1)** and IGKV1D-39 **(I2)**. **(J)** Validation of OS survival analysis in Kaplan-Meier plotter online sites for FBLN5 **(J1)**, TNFRSF14 **(J2)**, and SUSD3 **(J3)**.

The impact of 101 TME-key DCGs on the clinical prognosis of BRCA was also explored. A total of six prognostic marker genes, i.e., *COX7A1*, *ROBO3*, *FBLN5*, *IGKV1D-39*, *SUSD3*, and *TNFRSF14*, were hereby identified by univariate Cox regression analysis, LASSO regression (Figures 4A, B), and multivariate Cox regression analysis (Figure 4C), and a risk model was correspondingly constructed. The formula of the risk model is as follows: Risk Score = (−0.694 * expression level of TNFRSF14) + (−0.131 * expression level of SUSD3) + (0.517 * expression level of COX7A1) + (0.967 * expression level of ROBO3) + (−0.407* expression level of FBLN5) + (−0.341* expression level of IGKV1D-39).

All cases were divided into the high-risk and low-risk groups based on the median value of the risk score. According to Kaplan–Meier analysis, the survival curves of the high-risk patients were significantly lower than those of the low-risk patients (Figures 4D, E). Additionally, the AUCs based on the TCGA training cohort, TCGA test cohort, and GSE31448 cohort for 1-year, 3-year, and 5-year periods are shown in Figure 4F.

A study was conducted to correlate prognostic models with the clinical characteristics of BRCA based on the Wilcoxon rank-sum test. Higher risk scores were found in patients of advanced age (Age ≥ 65) (Figure 4G1). In menopause, patients in the post-menopause stage were exposed to a significantly higher risk than patients in the pre-menopause stage (Figure 4G2). In neoplasms, patients with tumors had a significantly higher risk score than that in those who were tumor-free (Figure 4G3). Among the different races, the risk score of white people was significantly higher than that of black people and African Americans (Figure 4G4). In the procedure, patients with modified radical mastectomy had significantly higher risk scores than those with lumpectomy and simple mastectomy (Figure 4G5). In the T-stage, patients in T4 were exposed to a significantly higher risk than other patients (Figure 4G6).

K–M survival analysis of TCGA-BRCA, GSE31448 cohort, and Kaplan–Meier plotter showed that among the six prognostic genes, *FBLN5*, *IGKV1D-39*, *SUSD3*, and *TNFRSF14* were of great significance in at least two datasets. *FBLN5*, *IGKV1D-39*, *SUSD3*, and *TNFRSF14* were significant in TCGA-BRCA cohort (Figure 4H). In the GSE31448 cohort, IGKV1D-39 and SUSD3 survival reached significance (Figure 4I). Kaplan-Meier plotter results present significant survival for FBLN5, SUSD3, TNFRSF14, and ROBO3 (Figure 4J). Currently, *FBLN5*, *SUSD3*, and *TNFRSF14* have been reported as prognostic markers for BRCA (Mohamedi et al., 2016; Aushev et al., 2018; Chen et al., 2022), and *IGKV1D-39* is a new potential BRCA prognostic marker discovered here.

### 3.4 Effect of different patient risks and IGKV1D-39 expression on BRCA TME

**FIGURE 5:**
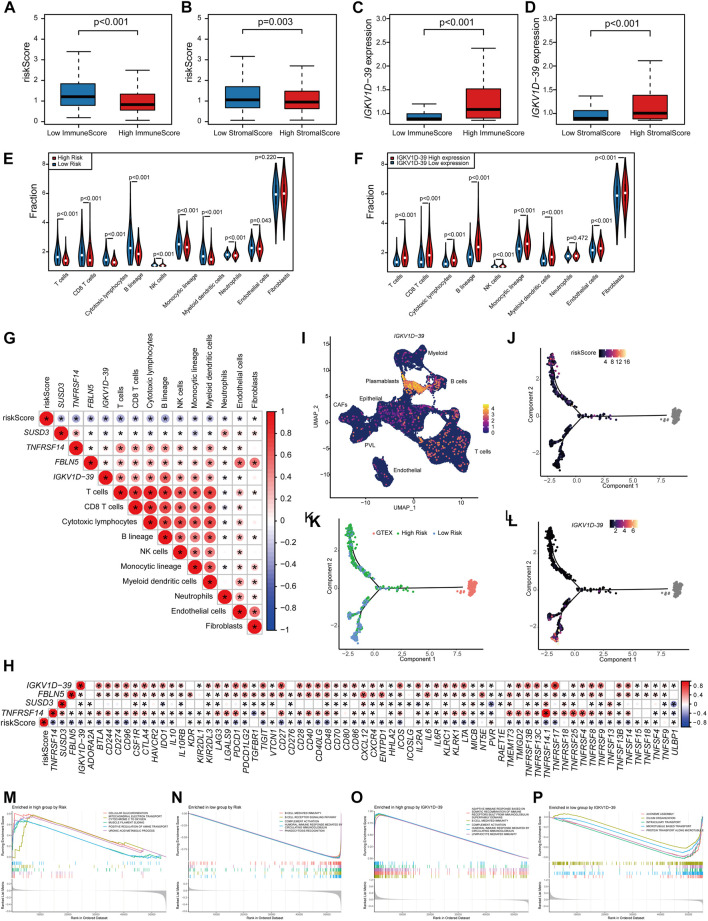
Prognostic model and prognostic markers with microenvironmental correlations: **(A,B)** The risk score was significantly correlated with ImmuneScore **(A)** and StromalScore **(B)**. **(C,D)** The IGKV1D-39 expression was significantly correlated with ImmuneScore **(C)** and StromalScore **(D)**. **(E,F)** MCPcounter calculations of immune cell infiltration levels of the 10 immune cell subgroups in the high–low risk group **(E)** and the high–low IGKV1D-39 expression groups **(F)**. **(E,F)** Differences in immune cell infiltration levels of the 10 immune cell subgroups calculated by MCPcounter in the high–low risk groups **(E)** and the high–low IGKV1D-39 expression groups **(F)**. **(G)** Correlation analysis of risk scores, prognostic genes, and the level of immune cell infiltration. Using Pearson calculations, *p* < 0.05 reached significant. **(H)** Correlation between risk scores, prognostic genes, and immune inhibitors and stimulators. Using Pearson calculations, *p* < 0.05 reached significance. **(I)** Cell clustering UMAP plot of BRCA-scRNA showing specifically high expression in the IGKV1D-39 gene in plasmablasts. **(J–L)** Risk scores **(J)**, risk grouping **(K)**, and the expression of IGKV1D-39 **(L)** in TCGA-BRCA patients are shown on the TME trajectory. **(M, N)** GSEA plot in high- and low-risk groups. The top five pathways with *p* < 0.05 and the highest NES values. **(M)** Upregulated pathways in high-risk patients. **(N)** Downregulated pathway in high-risk patients. **(O,P)** GSEA in the high–low IGKV1D-39 expression groups. The top five pathways with *p* < 0.05 and the highest NES values. **(O)** Upregulated pathways of the highly expressed IGKV1D-39 group. **(P)** Downregulated pathways of the highly expressed IGKV1D-39 group.

The relationship between the risk scores and TME of the patients, and the specific role played by the prognostic marker IGKV1D-39 in the TME was further investigated. Risk scores and IGKV1D-39 expression in TCGA-BRCA patients were found to be significantly different from their ImmuneScore and StromalScore, which are, indeed, lower in high-risk patients (Figures 5A, B). Analysis of immune cell infiltration levels showed that BRCA high-risk patients had fewer relative immune cell types (Figure 5E), which was significantly negatively correlated with the relative immune cell proportion (Figure 5G). In the correlation analysis with immune-related gene expression, the patient risk was found to be significantly and negatively correlated with the vast majority of immune inhibitors and immune stimulators (Figure 5H). IGKV1D-39 expression was negatively correlated with patient risks, and patients with a higher IGKV1D-39 expression had higher ImmuneScore and StromalScore (Figures 5C, D). Additionally, they also had a significant positive correlation with the proportion of multiple immune cells (Figures 5F, G), with the highest correlation in the B lineage (0.561). IGKV1D-39 was specifically highly expressed in the plasmablasts of BRCA-scRNA (Figure 5I), and its expression was significantly and positively correlated with most immune inhibitors and stimulators (Figure 5H). On the TME differentiation trajectory, the risk scores of these patients were elevated toward the direction of tumor progression (Figure 5J), and more of those in the high-risk group were distributed in the branch in the direction of tumor progression (Figure 5K). However, IGKV1D-39 was more expressed in the lower branches that favored stromal cell survival (Figure 5L).

GSEA pathway enrichment analysis found that the risk of patients upregulated energy metabolism, positive regulation of amine transport, and regulation of cell morphology, thereby possibly promoting BRCA proliferation and metastasis, and immune-related pathways were downregulated (Figures 5M, N). The expression of IGKV1D-39 upregulated immune-related pathways and downregulated pathways related to cell division and proliferation, such as chromosome segregation and vascular transport function. In this case, the *IGKV1D-39* gene might play an important immune role in BRCA TME and inhibit the activity of cancer cells (Figures 5O, P). IGKV1D-39, as a potential prognostic marker for BRCA, provides a new reference for the therapeutic target and prognosis of BRCA.

## 4 Discussion

Different stromal cell proportions in the TME affect tumor progression, and global changes in cell proportions reveal the direction of tumor development or even affect patient survival and prognosis, making it necessarily important to understand the cellular fractions in the TME and their phenotypes, so as to better understand the mechanisms of cancer progression and immunotherapeutic response.

CorDelSFS identifies genes associated with the stromal cell and immune cell proportions, and possesses a lower RMSE than other feature selection methods. Herein, the suitability of the characteristics of the learning model for the present feature selection strategy was analyzed, and the simplest classical linear regression model was found to be the most suitable for the feature selection strategy, followed by SVR, which was speculated to be related to the good robustness of linear regression. Neural networks might be more suitable for the prediction of rather large samples, such as image recognition.

The interaction between tumor cells and stromal cells leads to continuous changes in their abundance and function. Previous studies have overlooked the “dynamic” and “continuous” changes in cell proportions. Changes in cell abundance during the dynamic development of tumors and after certain critical biological events have been rarely studied. The TME-key genes identified by CorDelSFS are related to the proportion of stromal cells in different tumor states and can therefore reflect changes in cell proportions throughout tumor development. We constructed a pseudo-temporal ordering of tumor microenvironment development based on pseudo-temporal analysis. The process of TME changes is divided into two branches, with the upper branch developing in a direction favorable to tumor cells, with a low abundance of stromal cells, and the lower branch developing in a direction unfavorable to tumor cells, with a high abundance of stromal cells. Therefore, we have effectively simulated the dynamic process of the impact of TME cells on tumor development. Importantly, by combining CCEN and TME developmental trajectories, the plasmablast-specific IG gene subnetwork has contributed to the development of BRCA TME through adaptive immune responses toward branches favoring good patient prognosis. The immune gene *CXCL9* is a core gene (Figure 2K) that is co-expressed with IG genes and may play a key regulatory role. In addition, *IGKV1D-39* in the prognostic model constructed in TME-key DCGs is a newly identified prognostic marker of BRCA specifically expressed in plasmablasts.

**FIGURE 6:**
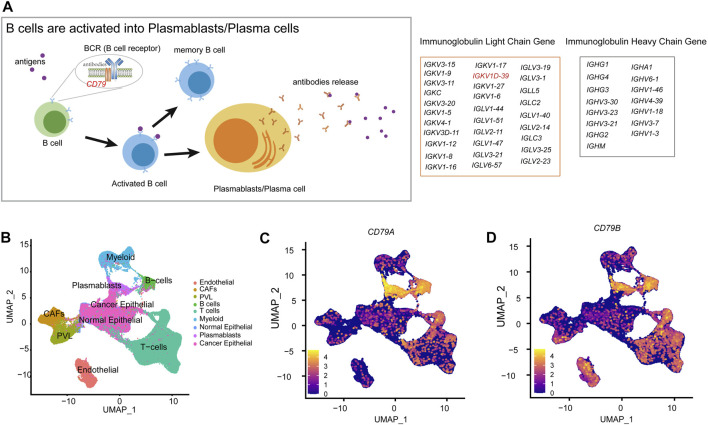
Molecular mechanisms of B-cell differentiation into plasmablasts. **(A)** Schematic diagram of the activation of B cells into plasmablasts. **(B)** Annotation of each cell type in the UMAP clustering map of BRCA-scRNA. **(C)** UMAP plot showing the expression of the *CD79A* gene in different cell types. **(D)** UMAP plot showing the expression of the *CD79B* gene in different cell types.

The role of B cells has been actually underestimated. However, B cells and antibodies matter considerably in the antitumor immune response (Zitvogel and Kroemer, 2015; Sharonov et al., 2020). The density of B cells and mature tertiary lymphoid structures (TLSs) is a major predictor for the response to immunotherapy (Engelhard et al., 2021). The presence of antibody-secreting cells and TLSs in the TME is generally associated with a favorable clinical prognosis (Petitprez et al., 2020; Meylan et al., 2022). Furthermore, plasmablasts are activated by B cells and exercise adaptive immune functions, while B-cell receptor (BCR) is a transmembrane protein on the surface of B cells, composed of CD79 and immunoglobulins, which will differentiate into plasmablasts after antigenic stimulation (Figure 6A). Then, plasmablasts can proliferate and differentiate into plasma cells in a short period of time and produce a large number of antibodies, which can be used to guide the immune system in producing correct immune responses to different types of foreign invaders encountered (Market and Papavasiliou, 2003). In BRCA-scRNA, *CD79A* and *CD79B* are specifically highly expressed in B cells and plasmablasts (Figures 6B–D), while plasmablasts secrete a large number of immunoglobulins in response to the variable TME, including IGKV1D-39 (Figure 5I). Additionally, the adaptive immune and membrane invagination pathways, which are significantly enriched in TME-key DCGs, are likewise most active in plasmablasts. More importantly, the adaptive immune function exerted by plasmablasts affects the state of the entire immune microenvironment and the process of the tumor, thereby resulting in a favorable patient prognosis. In this case, plasmablasts are important in BRCA by secreting large amounts of antibody-like immunoglobulins. Many researchers have investigated the role of antibody-like immunoglobulins in the antitumor process (Lacombe et al., 2022), and natural antibodies remind the adaptive immune system of the presence of transformed cells during early tumorigenesis (Rawat et al., 2022). Early neoantigen recognition and initiation of adaptive immunity require immune surveillance by natural IgM (Atif et al., 2018). Moreover, allogeneic IgG combined with dendritic cell stimulation induces antitumor T-cell immunity (Carmi et al., 2015). A new study by Mazor et al. has recently demonstrated that the immune system of cancer patients can produce antibodies against tumors (Mazor et al., 2022). However, antigenic specificity and the function of tumor-infiltrating B cells remain largely unknown, and natural antitumor antibodies show great potential for adjuvant immunotherapy. They hereby discovered that the BRCA prognostic marker IGKV1D-39, as a component of the immunoglobulin light chain, participates in the anti-tumor process through adaptive immunity, and may contribute to the study of unknown targets on the surface of tumor cells, thus providing new ideas for the clinical prognosis of BRCA and the development of immunotherapy drugs.

However, the present study is also subject to some limitations. Due to the complexity of the feature selection algorithm, only DEGs can be used for identification, with other important genes as well as co-expression patterns possibly overlooked. Inadequate sample size and incomplete information on the TME in BRCA patients may result in the incompleteness of the information on the development of the TME trajectory, and some key information may be lost.

## Data Availability

Publicly available datasets were analyzed in this study. This data can be found here: https://www.ncbi.nlm.nih.gov/geo/query/acc.cgi?acc=GSE31448; https://portal.gdc.cancer.gov/projects/TCGA-BRCA; https://singlecell.broadinstitute.org/single_cell/study/SCP1039/.
