# Supplementary figures and images for: Identification of stromal cell proportion-related genes in the breast cancer tumor microenvironment using CorDelSFS feature selection: implications for tumor progression and prognosis
Source: Front Genet. 2023 Jul 27;14:1165648. doi: 10.3389/fgene.2023.1165648 (PMC10421750; doi:10.3389/fgene.2023.1165648)

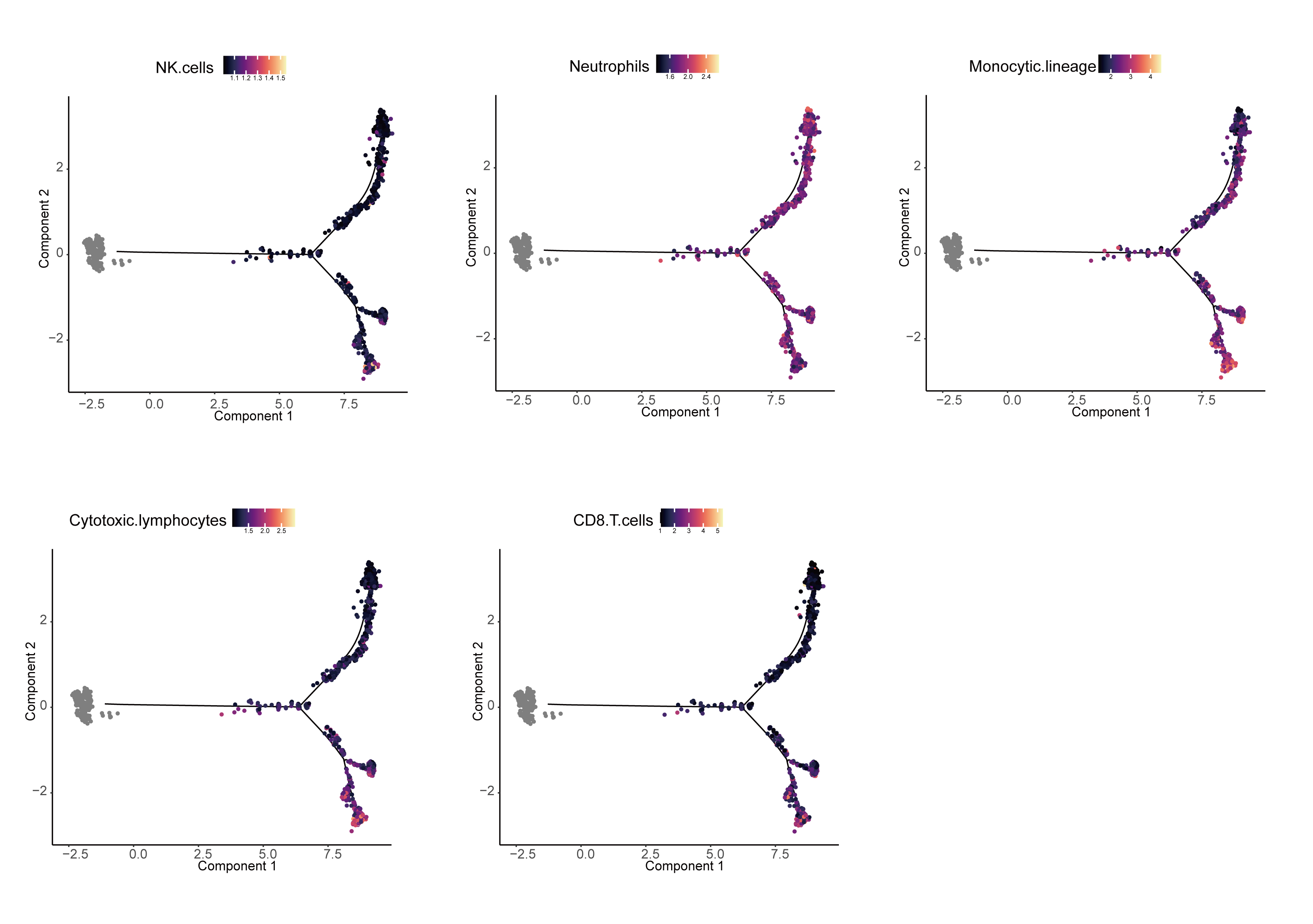

Supplement: Supplementary file 3 [file Image1.JPEG]
