# Supplementary material for: Identification of stromal cell proportion-related genes in the breast cancer tumor microenvironment using CorDelSFS feature selection: implications for tumor progression and prognosis
Source: Front Genet. 2023 Jul 27;14:1165648. doi: 10.3389/fgene.2023.1165648 (PMC10421750; doi:10.3389/fgene.2023.1165648)
Supplement: Supplementary file 8 [file DataSheet1.DOCX]

Supplementary Material

Bulk and single-cell multi-omics analysis combined with machine learning provided the view of dynamic trends in the tumor microenvironment in BRCA development and a novel prognostic biomarker

Sicheng Guo^1, †^, Yuting Ma^2, †^, Xiaokang Li^1^, Xiaogang He^1^, Yuan Hu^1,*^, Zheming Yuan^1,*^

*** Correspondence:** Yuan Hu: hy410302@163.com; Zheming Yuan: zhmyuan@sina.com

# Supplementary Data

Supplementary Material should be uploaded separately on submission. Please include any supplementary data, figures and/or tables.

Supplementary material is not typeset so please ensure that all information is clearly presented, the appropriate caption is included in the file and not in the manuscript, and that the style conforms to the rest of the article.

# Supplementary Figures and Tables

For more information on Supplementary Material and for details on the different file types accepted, please see [here](https://www.frontiersin.org/guidelines/author-guidelines#supplementary-material).

## Supplementary Figures


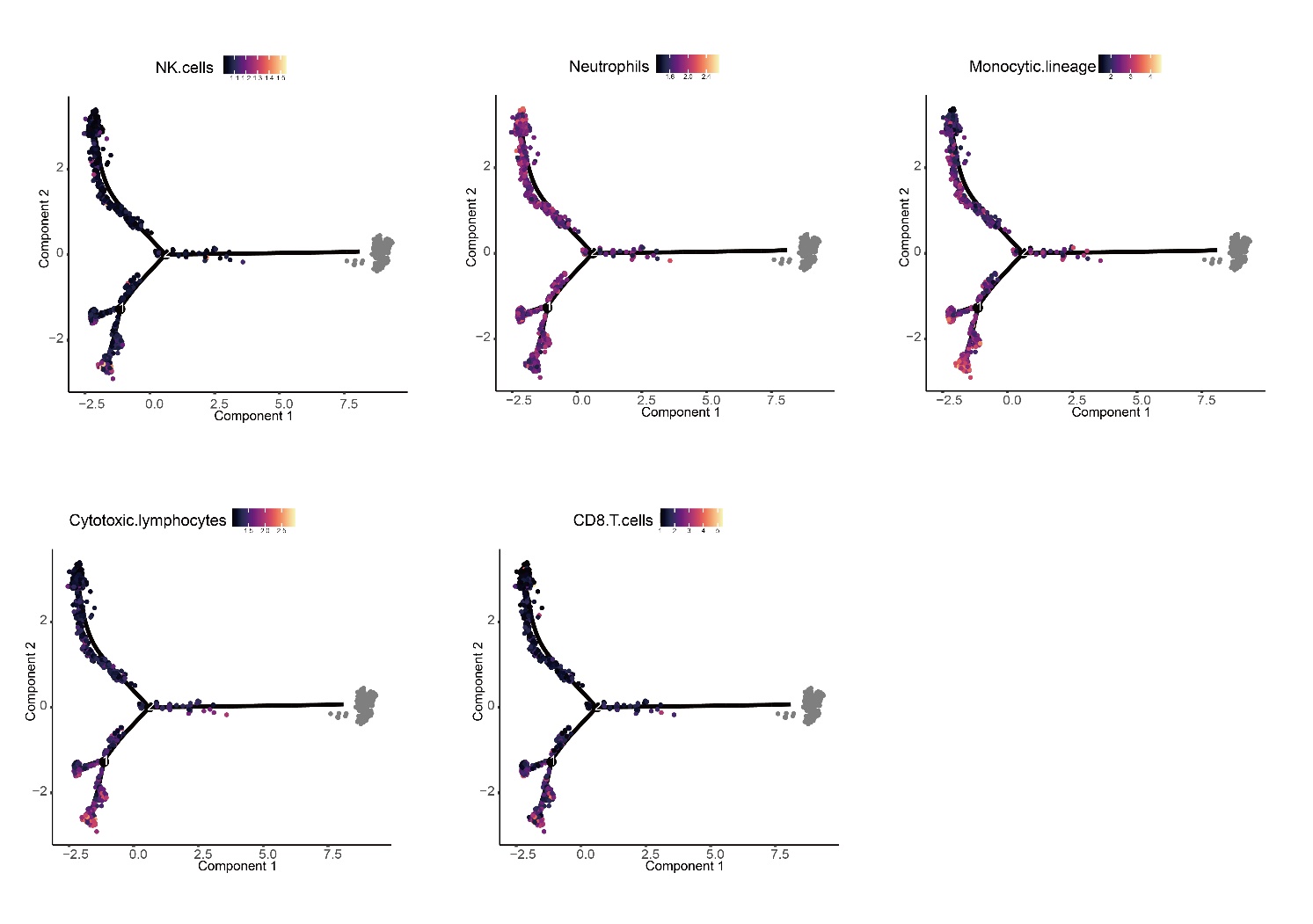


**Supplementary Figure 1.** Changes in the relative content of different types of infiltrating cells on the TME trajectory.

## Supplementary Tables
